# Wassermann Reaction among Insane Negroes

**Published:** 1914-05

**Authors:** 


WASSERMANN REACTION AMONG INSANE NEGROES. R. R. Ivey of Tuscaloosa, Ala., *N. Y. Med. Rec.*, Oct. 18, 1913, obtained 102 positive, 247 negative reactions in females, 90 positive and 267 negative reactions in males. A considerable variety of mental conditions is included, but it is significant that there were no negative reactions in the 17 cases of general paresis.

